# Sulfur Atom in its Bound State Is a Unique Element Involved in Physiological Functions in Mammals

**DOI:** 10.3390/molecules21121753

**Published:** 2016-12-21

**Authors:** Shin Koike, Yuki Ogasawara

**Affiliations:** Department of Analytical Biochemistry, Meiji Pharmaceutical University, 2-522-1 Noshio, Kiyose, Tokyo 204-8588, Japan; skoike@my-pharm.ac.jp

**Keywords:** bound sulfur, hydrogen sulfide, oxidative stress, persulfide, polysulfides

## Abstract

It was in the 1950s that the term polysulfide or persulfide was introduced in biological studies. The unfamiliar term “sulfane sulfur” sometimes appeared in papers published in the 1970s, and was defined in the review article by Westley in 1983. In the article, sulfane sulfur is described as sulfur atoms that are covalently bound only with sulfur atoms, and as this explanation was somewhat difficult to comprehend, it was not generally accepted. Thus, in the early 1990s, we redefined these sulfur species as “bound sulfur”, which easily converts to hydrogen sulfide on reduction with a thiol reducing agent. In other words, bound sulfur refers to a sulfur atom that exists in a zero to divalent form (0 to −2). The first part of this review focuses on the fluorescent derivatization HPLC method—which we developed for measurement of bound sulfur—and explains the distribution of bound sulfur and the hydrogen sulfide-producing ability of various tissues, as clarified by this method. Next, we discuss diverse physiological functions and involvement of polysulfide, a typical type of bound sulfur, in the redox regulation system. Additionally, we also address its possible physiological role in the central nervous system, based on its action of scavenging reactive carbonyl compounds.

## 1. Introduction

**Figure 1:**
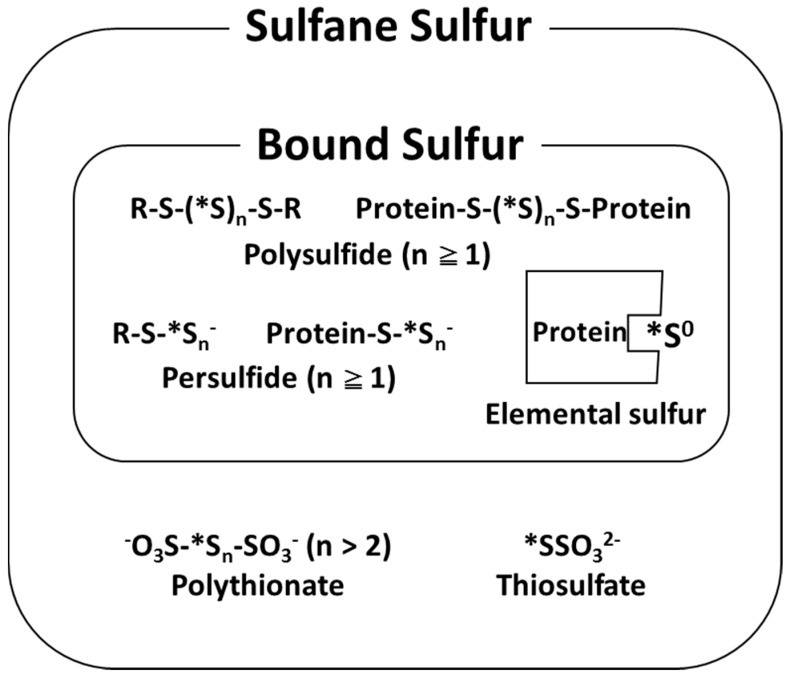
Definition of sulfane sulfur and bound sulfur. * sulfane sulfur.

In 1993, we defined sulfur atoms releasable as a hydrogen sulfide ion (HS^−^) by reducing agents, such as dithiothreitol (DTT), as “bound sulfur” [1]. Bound sulfur corresponds to the sulfur on the inner part of polysulfide and that on the outer part of persulfide (Figure 1).

Conventionally, hydrogen sulfide (H_2_S) generated in mammalian tissues is likely to be stored in its reduced state [2]. However, the physiological significance of bound sulfur was not fully understood for a long time. On the other hand, H_2_S rapidly became the focus of attention as an in vivo sulfur species after 1996. For example, it has been reported that endogenous H_2_S functions as a neuromodulator in the brain [3]. Thereafter, many studies of the signaling by H_2_S have been reported [4]. However, there are still a lot of unknown factors in terms of the mechanism of H_2_S, a gaseous signaling molecule, in biological tissues. Since the concentration of H_2_S present in vivo is significantly lower in comparison to that used in experiments, there were doubts regarding contribution of other sulfur species to the observed phenomenon. Accordingly, some physiological functions of bound sulfur, which were recognized as H_2_S storage, have become widely known lately, and many notable study reports have been presented in recent few years [5,6]. In this article, we describe the measurement method of bound sulfur, and its state of existence and physiological functions revealed through the application thereof, with focus on our novel findings.

## 2. Analysis and Characterization of Bound Sulfur

Quantitative determination of “sulfane sulfur” has been conventionally carried out using a reaction called cyanolysis [7]. This method causes a reaction between a cyanide ion and sulfane sulfur, resulting in the formation of a thiocyanate ion, which in turn, forms a complex with a trivalent iron ion. Finally, a colorimetric assay is used to determine the quantity of complexes of sulfane sulfur. However, the effects of cyanolysis cannot be adapted to thiosulfate and polythionate [7], which contain sulfane sulfur under usual reaction conditions, and this was one reason why the definition of sulfane sulfur had been obscure. Therefore, we clearly defined this reactive sulfur atom as “bound sulfur”, which is potentially released as HS^−^ with reducing agents such as DTT [1], as described above. Although the varieties of bound sulfur in biological tissues and their different reactivities, according to differences in form, are just being understood from recent biochemical studies, we have been aware of these matters since the beginning of our research in the 1990s. In other words, it is essential to carefully measure each of the low molecular-type sulfur (hydrogen polysulfide, cysteine persulfide, etc.) and high molecular-type sulfur (protein persulfide, elemental sulfur bound to protein, etc.) (Figure 1). Thus, we developed a highly sensitive and selective analysis method in which fluorescent derivatization of HS^−^ to thionine is performed with *p*-phenylenediamine and FeCl_3_, followed by separation and fluorescence detection with an HPLC system [8]. This method is the first in which free sulfide, acid-labile sulfur, and bound sulfur were simultaneously determined, and is at least 100 times more sensitive than the methylene blue colorimetric method, approximately five times more sensitive than the monobromobimane derivatization method, and comparable to the gas chromatography (GC)–flame photometric detector (FPD) method. Furthermore, the thionine–HPLC method, which can specifically measure HS^−^, was improved in combination with the pretreatment method [9], using continuous flow gas dialysis, and sequential procedures were applied in measuring the bound sulfur in human serum to demonstrate the ubiquitous existence of bound sulfur [1]. As a result, when bound sulfur was measured in healthy human serum (*n* = 10), the results were similar in males and females with concentrations of 1.16 ± 0.09 μM (*n* = 5) and 1.07 ± 0.18 μM (*n* = 5), respectively. The measured serum bound sulfur was significantly lower than the sulfane sulfur content (approximately 100 nmol/g) measured using the modified cyanolysis method by Westley et al. [10], but was closer to the protein bound sulfur content obtained by Sörbo et al. with the GC–flame ionization detector (FID) method, using *n*-tributylphosphine as a derivatization reagent [11]. Recently, bound sulfur species in the tissue have been measured using the headspace GC method [12]. However, there is room for improvement in this method: distinction of acid-labile sulfur and bound sulfur, and improvements in the measuring system to obtain the true values of bound sulfur species.

Next, we discuss the analytical method of polysulfide and persulfide using monobromobimane (mBB) as a labeling agent. In recent years, mBB has frequently been used in the quantitative determination of H_2_S [13]. We labeled polysulfide with mBB and attempted to carry out qualitative and quantitative assessment using LC–MS/MS and LC–fluorescence detection [14]. When examining the sample of a mouse brain, which was fluorescently labeled with mBB, using an independently developed method to measure polysulfide, endogenous H_2_S_n_ (*n* ≥ 2) was not found. However, by adding 3-mercaptopropionic acid (3MP) to a mouse brain cell suspension and incubating it for 15 min in 37 °C, the H_2_S level increased drastically, together with formation of a trace H_2_S_3_. In addition, when a similar experiment was conducted using a 3-mercaptopyruvate sulfurtransferase (3MST)-deficient mouse brain suspension cell, the alterations in H_2_S and H_2_S_3_ levels were not observed [14].

Based on the above results, it was suggested that in the brain, H_2_S and H_2_S_n_ are primarily produced through the pathway involving 3MST. Furthermore, modifying the separation conditions in the LC–MS/MS analysis allowed identification of H_2_S_2_ and H_2_S_4_, which could not be confirmed by the LC–fluorescence method. Unfortunately, it is extremely hard to accurately determine the quantity of hydrogen polysulfide (H_2_Sn, *n* ≥ 2), such as H_2_S_2_ and H_2_S_3_, using LC–MS/MS analysis. Although stock solution of each polysulfide is comparably stable in weak alkaline and hypoxic conditions, they gradually convert to various polysulfides in a solution, resulting in a pH-dependent state of equilibrium [5]. Therefore, even if stable polysulfide isotopes containing ^34^S are prepared individually, each form seems to be naturally exchanged in a neutral solution, making it extremely challenging to maintain a standard solution containing a specific polysulfide. Such characteristics of each type of polysulfide make quantitative determination of each polysulfide difficult, despite utilization of LC–MS/MS analysis.

Meanwhile, similar to polysulfide, with low-molecular persulfide (glutathione (GSH) persulfide, cysteine persulfide, etc.), mutual exchange reactions occur in the standard solution at a physiological pH, thereby making quantitative determination quite difficult [15]. After fluorescent labeling with mBB, the detection could be performed with the LC–fluorescent method or LC–MS/MS. In recent studies, along with observation of the persulfuration of cysteine residues in protein adjusting functions, detection of persulfurated protein (namely, high-molecular type persulfide) is being attempted. In recent reports, the biotin switch technique [16,17] and the tag switch method [18] have been used to measure persulfides. In the biotin switch method, thiol is specifically blocked with methyl methanethiosulfonate (MMTS), followed by detection of the reaction between protein persulfide and *N*-(6-(biotinamido)hexyl)-3′-(2′-pyridyldithio)-propionamide (biotin–HPDP). However, the selectivity of MMTS in differentiating between thiol and persulfide is being questioned, and it is possible that high-molecular persulfide is not being accurately measured [19]. In the tag switch method, 2-methylsulfonyl benzothiazole (MSBT) is reacted with a protein cysteine residue and persulfide residue, followed by reaction with cyanide (CN)-biotin. Persulfide can be specifically detected by the difference in the reactivity of the nucleophilic material of the reaction product (CN-biotin). A limitation of this technique is that due to the low membrane permeability of MSBT, upon applying this measurement system to living cells, only a limited portion reacts with MSBT [19]. Recently, Cuevasanta et al. have developed a modified measurement system for high molecular-type persulfide [20]. Nagy et al. applied the modified method to viable cells and, according to their report, there are 11.6 ± 6.9 μg/mg high-molecular persulfides in mouse liver [19].

As described above, it is desirable to establish a system that not only determines the overall bound sulfur quantity, but also clarifies details such as the type of bound sulfur, where it functions in mammalian tissues, and its exact quantity. The point is that polysulfide and persulfide are interchangeable in physiological solutions and exist in a state of equilibrium (Figure 1). Therefore, it is unlikely to presume that they maintain a fixed form in biological tissues. In addition, there is concern that during sample preparation, specific polysulfide, persulfide, or H_2_S may be produced artificially. Currently, accurate identification, quantification, and visualization of bound sulfur within mammalian tissues are extremely hard, necessitating various improvements in the future.

## 3. Metabolic Pathways of H_2_S and Bound Sulfur

**Figure 2:**
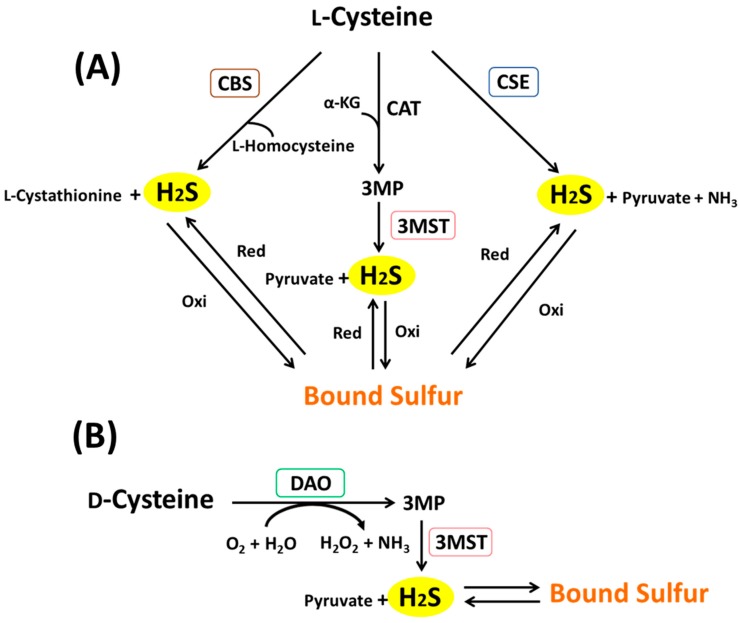
H_2_S and bound sulfur generation from l-cysteine (**A**) and d-cysteine (**B**) in mammalian tissues. CBS: cystathionine β-synthase; CSE: cystathionine γ-lyase, CAT: cysteine aminotransferase, MST: mercaptopyruvate sulfurtransferase, α-KG: α-ketoglutarate, DAO: d-amino acid oxidase, 3MP: 3-mercaptopyruvate, red: reduction; oxi: oxidation.

In mammalian tissues, H_2_S is produced through three enzymatic pathways with l-cysteine (cysteine) as a substrate: cystathionine γ-lyase (CSE), cystathionine β-synthase (CBS), and cysteine aminotransferase (CAT)/3MST [21,22] (Figure 2A). Additionally, it has been discovered that H_2_S is also produced when d-cysteine is added to mouse brain homogenate, instead of l-cysteine [23] (Figure 2B).

CSE, CBS, and CAT are pyridoxal phosphate (PLP)-requiring enzymes. Accordingly, a PLP-dependent enzyme inhibitor was added in a set of experiments; however, no effect was observed on H_2_S production from d-cysteine. Thus, existence of a fourth production pathway was hypothesized. It has been reported that in mammalian cells, α-keto acid is produced from d-amino acid by means of d-amino acid oxidase (DAO) [24]. Using the DAO inhibitor, we examined the 3MP and H_2_S production quantity, and discovered the involvement of DAO on the production of 3MP and H_2_S from d-cysteine in the mitochondrial fraction of mouse brain. These results also indicated, for the first time, that it is possible for d-cysteine to be converted to 3MP, which is an α-keto acid, by DAO [23]. In mice, DAO is expressed specifically in the cerebellum and kidneys. Therefore, it is suggested that d-cysteine is metabolized to 3MP by means of DAO, and when 3MP is metabolized by 3MST, H_2_S is produced in brain and kidney. In mammals, the intrinsic existence of d-cysteine has not been confirmed so far, and it is unknown whether this enzymatic pathway functions physiologically. However, in comparison to l-cysteine, which exhibits toxicity at a high concentration, the extent of d-cysteine toxicity is low, stirring hopes of medical application as an H_2_S-generating agent. Our experiments with mice have indicated the suppression of ischemic nephropathy through oral administration of d-cysteine, suggesting its potential in medical applications [23].

## 4. The Significance of Differentiating between Hydrogen Sulfide and Bound Sulfur

### 4.1. The Issues with Previous Studies

Studies spanning the past 20 years have gradually revealed the physiological functions and production pathways of H_2_S in biological tissues. Unfortunately, there are obvious ambiguities concerning the reaction mechanism of H_2_S at the molecular level. Snyder et al. proposed that a sulfur atom binds to the cysteine residue in a protein, producing the –SSH group, which regulates the protein function [16]. Snyder et al. also termed this phenomenon as “sulfhydration.” However, it is considered that *S*-sulfhydration should be called “persulfidation” [25]. In the present review, we refer to the reaction forming the –SSH group as “persulfidation.” There are doubts as to whether the persulfidation reaction occurs between H_2_S and cysteine thiol residue, both of which have valency number of −2. Also, it was reported that H_2_S alone can cause protein persulfidation by reacting with sulfenic acid [20,26,27]. In either case, it is appropriate to assume that the –SSH group is not produced from a nonenzymatic direct reaction between H_2_S and a cysteine residue. Furthermore, many studies have used a high concentration of sodium hydrosulfide (NaHS) as a H_2_S-generating agent. The background factor, in terms of the usage of such a high concentration of a H_2_S-generating agent (in the millimolar order), is the influence of early reports of values of several tens or hundreds micromolars of H_2_S in biological tissues [28,29,30]. Recently, based on the progress of analytical techniques and understanding of the issue concerning in vivo sulfur species, it has been clarified that the H_2_S concentration in biological tissues is several micromolar to nanomolar [31,32]. Therefore, it is likely that the H_2_S concentrations used in various studies so far differ markedly from the physiological value of H_2_S. In addition, commercially available NaHS includes many byproducts (different from sulfides), which could possibly have influenced the results observed in previous studies [33]. Moreover, even with the use of sodium sulfide (Na_2_S), a H_2_S-generating agent with a high level of purity, it has been indicated that the effects of bound sulfur that is potentially generated by immediate oxidation of H_2_S in the solution cannot be completely eliminated [34,35]. Thus, it is now presumed that part of the action attributed to H_2_S in these studies may have been due to different sulfur species. Consequently, bound sulfur, which so far has been considered as the storage form of H_2_S, is gaining much attention.

### 4.2. An Old and New Physiological Metabolite Called Bound Sulfur

In an earlier study, Stipanuk et al. stated that endogenously produced free sulfur atoms are stored in a reduced state [2]. Westley et al. defined the physiological storage forms of the reduced sulfur species as the sulfane pool [36,37,38,39]. Furthermore, they defined the minus divalent sulfur atom, which binds only to another sulfur atom, as “sulfane sulfur.” Specifically, it refers to the sulfur species that converts into a thiocyanate ion after undergoing cyanolysis. However, the form and quantity of sulfane sulfur in mammalian tissues has been obscure, to date. Consequently, we redefined sulfane sulfur, excluding thiosulfate, which does not release HS^−^ by DTT reduction, as bound sulfur, and developed a selective and differential method for quantitative determination of sulfide and bound sulfur in various mammalian tissues [1,8,40]. Ishigami et al. confirmed from their experiments of adding H_2_S to mouse brain homogenate that the added H_2_S is retained in the tissue as bound sulfur, indicating the possibility that bound sulfur is a storage form of H_2_S produced in tissues [41]. Moreover, the immunostaining method showed that in the mouse brain, 3MST is expressed in nerve cells, clarifying that H_2_S and its storage form (namely, bound sulfur) are produced from the CAT/3MST pathway [23]. In the enzymes involved in producing H_2_S and bound sulfur, little activity of CSE was detected in the brain, while CBS is clearly expressed in glial cells [42]. Thus, in the mouse brain, it is likely that the bound sulfur in nerve cells is produced from 3MST, and the bound sulfur in glial cells is produced from CBS. However, in terms of the localization of 3MST, Nagahara et al. reported that bound sulfur is expressed in glial cells in the rat brain [43]. Recent reports have also shown 3MST expression in astrocytes, a type of glial cells, in the rat brain [44]. In understanding the physiological functions of bound sulfur in the brain, it is imperative to show the localization of the enzyme produced. Therefore, it is desired that the localization of 3MST in the brain should be clarified in the future. Although a few unclear points persist, the existence of bound sulfur and its production system in mammals have been studied intensively, in spite of studies on its physiological functions being limited to those indicating inhibitory effects for thiol-dependent enzymes [45]. The recent few years witnessed an unexpected increase in the number of studies referring to the physiological functions of bound sulfur and its reaction mechanism, after approximately 20 years since first defining bound sulfur. The common factor in these reports is the persulfidation of the cysteine residue in proteins (phosphatase and tensin homolog (PTEN), Kelch-like ECH-associated protein 1 (Keap1), transient receptor potential cation channel, subfamily A, member 1 (TRPA1), etc.) by bound sulfur, fulfilling its function through the mechanism of signal transduction [34,46,47]. There have also been reports of persulfidation of the cysteine residue in glyceraldehyde 3-phosphate dehydrogenase (GAPDH) by polysulfide, causing decreased activity [48]. As a result, studies on bound sulfur have been rapidly performed, and new functions have been determined in the past few years. In the following section, we elaborate on the physiological functions of bound sulfur that have recently been demonstrated.

### 4.3. Cytoprotective Effect against Oxidative Stress

**Figure 3:**
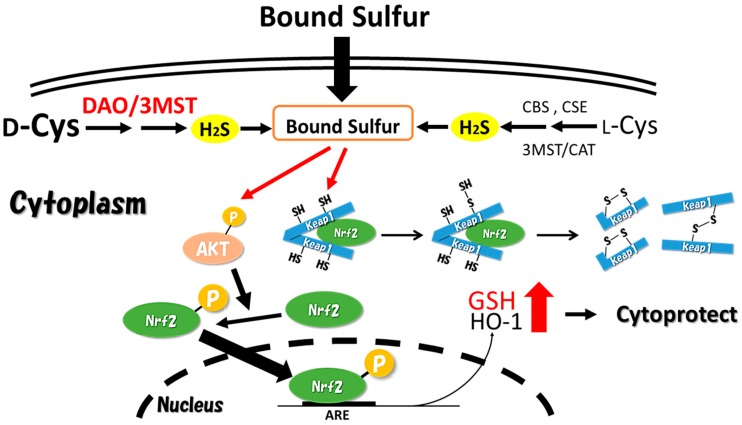
Bound sulfur activates the nuclear factor erythroid related factor 2 (Nrf2) system through a structural change of Kelch-like ECH-associated protein 1 (Keap1) and promotion of the AKT/phosphoinositide 3-kinase (PI3K) signaling pathway. ARE: antioxidant response element; GSH: glutathione; HO-1: heme oxygenase 1.

It has been revealed that oxidative stress is closely intertwined with various diseases in mammals. In the central nervous system, its correlation with Parkinson’s disease and Alzheimer’s disease has been suggested [49,50]. The functions of nuclear factor erythroid related factor 2 (Nrf2) were clarified, by Itoh et al. in 1997, as factors in controlling intracellular antioxidative gene clusters. Nrf2, a transcription factor, is captured by Keap1 during non-stress conditions in the cytoplasm [51]. Keap1, an adapter protein for Cullin3 type E3 ligase, is decomposed by proteasomes after the ubiquitination of Nrf2 captured by Keap1. However, when the cells are exposed to electrophilic materials or reactive oxygen species, Nrf2 in the cytoplasm avoids being captured by Keap1 and translocates into the nucleus. The Nrf2 translocated into the nucleus forms a heterodimer with small Maf proteins (sMaf), binds with antioxidant response elements (AREs), and induces the expression of various antioxidative gene clusters existing in downstream AREs. The Keap1/Nrf2 system is an ingenious defense mechanism against oxidative stress in the cells. There are several reports of H_2_S-induced nuclear translocation of Nrf2 [52,53], and Hourihan et al. suggested the induction mechanism [54]. In their study, Hourihan et al. added NaHS as a H_2_S donor to cultured cells and proved that H_2_S directly modifies Keap1 cysteine residues and induces nuclear translocation of Nrf2. In addition, it was concluded that this mechanism is attributed to H_2_S directly modifying the Keap1 cysteine residue, and the structural change of Keap1 due to the H_2_O_2_ generated from H_2_S oxidizing the Keap1 cysteine residue [54]. However, as Toohey pointed out, in actuality, it is unlikely that H_2_S reacts nonenzymatically with the cysteine residue [55]. Moreover, the extremely high NaHS concentration of 400 μM added in their study is not physiologically relevant for such a persulfidation reaction to occur in the cell. Prior to this report [54], Yang et al. suggested a mechanism of H_2_S forming the –SSH residue in Keap1, structurally disturbing the ubiquitination of Nrf2 with Keap1 [20]. However, no experimental evidences of the phenomena were shown in the report, thus their assertion is only a hypothesis. We have also attempted to clarify the intranuclear translocation of Nrf2 by bound sulfur, and resolved the mechanism, as shown in Figure 3. On adding a typical bound sulfur, called sodium tetrasulfide (Na_2_S_4_) to neuroblastoma cells, we found that Nrf2 accumulated in the nucleus [46]. Furthermore, with the intranuclear accumulation of Nrf2 after the polysulfide treatment, the levels of intracellular reduced GSH and heme oxygenase-1 expression clearly increased, proving that polysulfide protects against oxidative stress. Secondly, we embarked on the clarification of this induction mechanism. We found that polysulfide modified the cysteine residue in Keap1 and changed the structure of Keap1 to form dimers. This phenomenon is attributed to the persulfidation of the cysteine residue in Keap1 by the reaction with polysulfide and the disulfide exchange reactions, which lead to Keap1 forming heterodimers with another protein, or homodimers with another Keap1 protein. Furthermore, it was revealed that polysulfide induced AKT phosphorylation, followed by the phosphorylation of Nrf2 by the phosphorylated AKT, resulting in translocation of Nrf2, which escaped capture by Keap1 in the cytoplasm and stably migrated into the nucleus. The AKT phosphorylation by polysulfide, which we observed, is estimated to be based on the inactivating effect of polysulfide against PTEN [34]. Greiner et al. clarified that when polysulfide is processed in HEK 293T cells, the two cysteine residues in the PTEN molecule are oxidized, forming disulfides, thereby inactivating the PTEN [34]. Thus, it is considered that polysulfide treatment promotes phosphorylation of AKT by phosphoinositide 3-kinase (PI3K). In summary, we proved that polysulfide caused structural changes in Keap1, and through the phosphorylation of Nrf2, the intranuclear translocation of Nrf2 is actively induced (Figure 3).

Induction of the intranuclear translocation of Nrf2 by polysulfide has been significantly confirmed by the treatment with low level of Na_2_S_4_. Therefore, the regulation of Nrf2 system by such bound sulfur is estimated to be a novel defense mechanism against oxidative stress in the cell. Additionally, it is presumed that the induction of intranuclear translocation of Nrf2 by H_2_S generation is caused via bound sulfur species derived from H_2_S. Recently, Ida et al. reported that GSH persulfide and cysteine persulfide exist in mouse tissues [56]. This study indicated that GSH persulfide has a stronger antioxidant potential in comparison to GSH. Thus, these results also support our finding that bound sulfur functions as an endogenous defensive factor against oxidative stress in mammals.

### 4.4. Physiological Action against Glycation Stress

**Figure 4:**
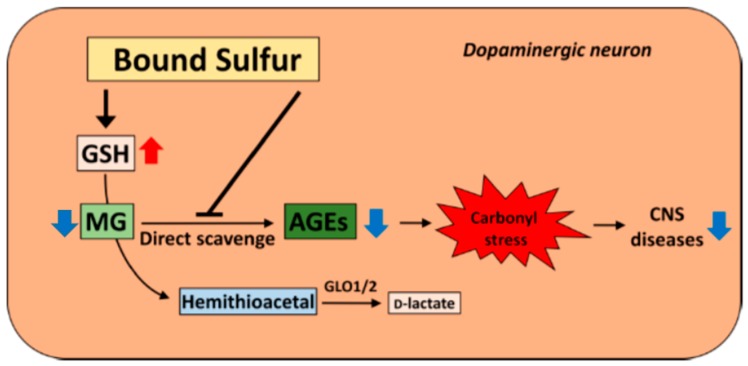
Proposed mechanisms of the protective effects of polysulfides against carbonyl stress in neuronal cells. MG: methylglyoxal; GLO: glyoxalase; AGE: advanced glycation end product, CNS: central nervous system.

Glycation (carbonyl) stress refers to the accumulation of advanced glycation end products (AGEs) in the cells. AGEs are produced when a protein is non-enzymatically modified by reactive carbonyl compounds (RCOs) produced from sugar and lipids [57]. Presently, the correlation between neurodegenerative diseases and glycation stress is attracting attention, and its relation to Alzheimer’s disease and Parkinson’s disease has also been suggested [58]. In addition, a subgroup of schizophrenic (SCZ) patients showing glycation stress has been reported. This patient group with so-called carbonyl stress-type schizophrenia (CS-type SCZ) exhibited a high level of pentosidine, which is a type of AGE, in the plasma, and low vitamin B_6_ levels in the serum [59]. Furthermore, in this CS-type SCZ group, a few patients with 50% less activity of glyoxalase (GLO1)—a metabolizing enzyme of methylglyoxal (MG), a type of RCO—were identified. Accumulation of AGEs is caused by RCOs, and improvement in SCZ symptoms has been reported when pyridoxamine, which is a strong scavenger of RCOs, is orally administered to CS-type SCZ patients [60]. Therefore, an RCO scavenger is expected to have marked efficacy in CS-type SCZ and other diseases related to carbonyl stress. Chang et al. reported that H_2_S has the ability to eliminate MG-induced toxicity [61]. Apart from the previous study, we anticipated the possibility that polysulfide, not H_2_S, has scavenging ability against methylglyoxal. Using 1,2-diamino-4,5-methylene-dioxybenzene (DMB), which is a fluorescent labeling agent for α-keto acid, intracellular MG was measured using the fluorescent HPLC method [62]. Although the intracellular MG concentration increased in a dose-dependent manner on treatment with polysulfide, the increase in MG was suppressed. Addition of polysulfide to human neuroblastoma cells increased the level of intracellular GSH, which is indispensable for MG metabolism. Furthermore, pretreatment of cells with buthionine sulfoximine (BSO), an inhibitor of GSH synthesis, also failed to suppress the effect of polysulfide treatment on MG toxicity. Moreover, we demonstrated that polysulfide inhibited the formation of MG-induced modified protein in the nerve cells, but the inhibitory effect was not lost even with BSO pretreatment. These results indicate that polysulfide promotes GSH synthesis, and the subsequent increase in GSH concentration partially contributes to alleviating MG toxicity. Also, the existence of an inhibitory effect by bound sulfur through a different mechanism was also elucidated. We mixed MG with polysulfide in a phosphate buffer in order to determine the direct effect of polysulfide on MG-induced toxicity. The result showed concentration-dependent MG-eliminating ability, indicating that polysulfide can play a role as a direct scavenger against α-dicarbonyl compounds [63]. Thus, it seems that polysulfide acts through a GSH-dependent mechanism and direct scavenging reactivity to weaken glycation stress. Thus, polysulfide may be effective in the treatment of diseases of the central nervous system involving carbonyl stress, as MG causes strong carbonyl stress. Considering the existence of bound sulfur in brain, there is a possibility that bound sulfur takes part in the regulation of glycation in the nerve cells (Figure 4).

Moreover, there is a report that in the heart and liver of CSE-deficient mice, the bound sulfur level is lower than that in wild-type mice, and the carbonylated protein level increased in the CSE-deficient mice [64]. A study using CBS-deficient mice also showed an increase in carbonylated protein levels in the tissue of deficient mice compared to that in wild-type mice [65]. Accordingly, when the bound sulfur level is decreased, the subject is presumed to be more susceptible to glycation stress.

## 5. Conclusions

Among the sulfur species existing in mammalian tissues, bound sulfur, which was generally and virtually unknown, was found to have various physiological functions, rapidly gaining attention in the field of life science. It was gradually revealed that the primary mechanism in the physiology of bound sulfur is modification of the inherent protein function through persulfidation of cysteine residues in the protein. This phenomenon, known as persulfidation, is a reaction that is difficult to achieve with H_2_S directly. It has been contentiously clarified that many of previously reported physiological functions, which were attributed to H_2_S, may likely have been due to its oxidized form, bound sulfur species, such as polysulfide. In the meantime, it was assumed that H_2_S is produced in biological tissues, acting as a signal molecule. As mentioned previously, it is clear that polysulfide and persulfide exist interchangeably in mammalian tissues, and it is hypothesized that H_2_S is produced in the process of their interconversion. Consequently, research based on the inappropriate premise that H_2_S exists in the gaseous molecule in physiological conditions is undesirable. This ambiguous notion should be discarded in future studies of polysulfide, persulfide, and other bound sulfur species. We hope that studies of in vivo sulfur species will continue to progress with thoroughly correct understanding in the future.
